# The Relationships of Sleep Duration and Inconsistency With the Athletic Performance of Collegiate Soft Tennis Players

**DOI:** 10.3389/fpsyg.2022.791805

**Published:** 2022-03-24

**Authors:** Tianfang Han, Wenjuan Wang, Yuta Kuroda, Masao Mizuno

**Affiliations:** ^1^Graduate School of Health Sciences, Hokkaido University, Sapporo, Japan; ^2^Graduate School of Education, Hokkaido University, Sapporo, Japan; ^3^Department of Sport Education, Hokusho University, Ebetsu, Japan; ^4^Faculty of Education, Hokkaido University, Sapporo, Japan; ^5^Faculty of Health and Medical Care, Hachinohe Gakuin University, Hachinohe, Japan

**Keywords:** sleep duration, sleep inconsistency, serve, performance, agility

## Abstract

We evaluated the relationships of daily sleep duration and inconsistency with soft tennis competitive performance among 15 healthy collegiate soft tennis players (13 male, 2 female, mean age = 19.7 ± 0.8 years, height = 170.8 ± 7.3 cm, weight = 60.3 ± 5.6 kg, soft tennis experience = 8.7 ± 2.0 years). Sleep duration and inconsistency were determined by a 50-day sleep diary, which recorded sleep and wake times of sleep. Soft tennis athletic performance was evaluated by a service and baseline stroke accuracy test and the spider run test. Mean sleep duration was 7.4 ± 1.7 h. No correlation was found between long-term mean sleep duration and athletic performance. But inconsistency in sleep duration (SD of sleep duration) was inversely correlated with service score after controlling for soft tennis experience and sex (*r* = −0.56, *p* = 0.046). There was no significant relationship between sleep inconsistency and other athletic performance. This result indicates that reducing the instability of sleep duration (i.e., sleep regular hours) in the long-term may have a positive effect on soft tennis players’ service performance. Although participants’ current mean sleep duration (7.4 h) was not as sufficient as the recommendation in sleep extension experiments (9–10 h), it revealed the importance for athletes to maintain regular sleep in daily life.

## Introduction

Experimental evidence for sleep extension has accumulated and demonstrated the important role of sleep duration on athletic performance (Watson, 2017). Mah et al. (2011) revealed that basketball performance is improved by prolonged sleep duration. Specifically, 11 players were asked to lie on a bed 10 h every night for 5–7 weeks to evaluate the effect on their daytime performance. Compared to baseline, participants’ sprint time was 0.7 s shorter after sleep extension, and their shooting accuracy was improved: successful free throws increased 0.9 times and successful three-point field goals increased 1.4 times. The results also showed a faster timed on-court sprint following sleep extension. A more recent study that involved 12 collegiate tennis players, extending sleeping time up to 9 h for 1 week, improved the service accuracy from 36 to 42% (Schwartz and Simon, 2015).

Three additional experiments that examined sleep restriction revealed a lower service accuracy among collegiate tennis players (Reyner and Horne, 2013). Their first experiment involved 16 collegiate tennis players and compared a 5 h sleep (33% restriction) condition with a normal sleep condition. Researchers found that players’ service success (total 30 times) decreased about four times in both male and female players after sleep restriction. Their second experiment involved 12 collegiate tennis players and reduced normal sleeping time by 33% (the same as in the first experiment); however, participants then ingested 80 mg of caffeine before the performance measurement. Results showed that, although players’ service success (total 30 times) in the caffeine condition was better than in the restricted no caffeine condition, the success rate was still about three times lower than in the normal condition for both male and female players (Reyner and Horne, 2013). A third study conducted a randomized, counterbalanced, and crossover experiment to evaluate the effect of acute sleep restriction on sport-specific technical and athletic performance in male junior tennis players; compared to the control group, significant decreases were observed in serve accuracy and crosscourt shots after experiencing acute sleep restriction (sleep ≤ 5 h) the night before the test session (Vitale et al., 2021). In basketball, Filipas et al. (2021) also observed negative effects on free-throw after participants experienced sleep restriction and mental fatigue.

Clearly, laboratory studies have been demonstrating that sleep extension and restriction affects athletic performance among collegiate players. And systematic review also had determined that the sports requiring speed, tactical strategy, and technical skill were the most sensitive to sleep duration manipulations (Kirschen et al., 2020). Nevertheless, collegiate athletes have been reporting experiencing poor and insufficient sleep in reality (Mah et al., 2018). Further contributing factor presumed to affect athletic performance is sleep inconsistency, sometimes called “social jet lag.” This is defined by an inconsistency in sleep schedule and/or duration (Okano et al., 2019). Professional athletes usually experience fatigue and poor sleep and when they travel to countries in different time zones to participate in international competitions, therefore, inconsistency in sleep is considered unfavorable and thought to disrupt the synchrony of circadian rhythms. Not merely one night of impaired sleep but extreme sleep loss or accumulated sleep debt may have negative consequences, and affect subsequent sleep duration/quality, and performance (Knufinke et al., 2018b; Nedelec et al., 2018; Janse Van Rensburg et al., 2021). In Japan, collegiate students join the soft tennis team of university, participate training while studying university courses, and some of them become professional athletes after graduate. To our knowledge, there is a dearth of studies evaluating sleep duration and inconsistency with the athletic performance of collegiate sports players in more naturalistic settings (Irish et al., 2015).

Therefore, the present study examined the relationship between sleep duration, inconsistency and the athletic performance of collegiate players by employing naturalistic observational methods. Clarifying these relationships informs strategies to improve athletic performance. We hypothesized that we would find a negative correlation between sleep indices and athletic performance if participants showed insufficient and large inconsistencies in their sleep.

## Materials and Methods

### Participants

Twenty-seven healthy soft tennis players (23 male, 4 female, mean age = 19.9 ± 1.0 years, mean height: 170.8 ± 6.9 cm, mean weight: 61.1 ± 5.9 kg, experience = 7.5 ± 2.4 years) belonging to the Hokkaido University soft tennis team, who also had experience participating in the Hokkaido Soft Tennis Championship were recruited at the beginning. Players were requested to complete the Morningness-Eveningness Questionnaire (Horne and Ostberg, 1976) at the beginning, which identifies extreme morning or evening chronotypes (Morningness–Eveningness Questionnaire < 30 or > 70), and no players reported sleep problems. There were no extreme morning or evening chronotypes to exclude, but we did exclude potential participants who did not complete the performance test (*n* = 2) and had incomplete diaries (sleep diary < 50 days, *n* = 10). Finally, 15 participants both finished the 50-day sleep diary and performance test were included in analyses (13 male, 2 female, mean age ben = 19.7 ± 0.8 years, height = 170.8 ± 7.3 cm, weight = 60.3 ± 5.6 kg, soft tennis experience = 8.7 ± 2.0 years). Soft tennis differs from regular tennis in that it uses soft rubber balls instead of hard yellow balls. It is played primarily in Japan, and it was introduced to Europe in 2004. Participants had the procedures fully explained to them, along with their freedom to withdraw, signed consent forms, and voluntarily participated in the experiment without pay. Moreover, personal information was strictly protected. The experimental protocol was approved by the Hokkaido University Faculty of Education Ethics Committee (No. 17-37-02) and conducted according to the ethical standards described in the Declaration of Helsinki.

### Design and Procedures

#### Sleep Index

In the present study, participants were instructed to use a sleep diary (Yamanaka et al., 2010) to record their sleep duration for 50 days including the times they go to bed, get up, and nap. Sleep duration for 1 day was a sum of naps and night-time sleeping time (Schwartz and Simon, 2015). We used the raw data of mean sleep duration as well as intra-individual variability (i.e., standard deviation) of sleep duration in each participant. The intra-individual variability of sleep duration was used as the measure of sleep inconsistency (Okano et al., 2019).

#### Evaluation of Athletic Performance

Participants performed three tests: a service test, a baseline stroke test, and a spider run. Participants were instructed to eat and sleep as usual and avoid alcoholic drinks for 1 day before the performance test. To avoid the negative influence of menses, female participants were requested to record menstruation in their sleep diaries to determine a suitable day for their athletic performance test.

The performance test was conducted on the soft tennis team training courts (outdoor, clay) in Hokkaido University, test time was 8:30 a.m.-11:30 a.m. on October 21st, 2017. Participants used their soft tennis rackets and balls as usual (soft tennis ball and racket are different from tennis, but serve method and court are as same as tennis). The test protocol design consists of 10 min warming up, 40 min service test, 60 min baseline stroke test, 5 min rest, 10 min spider run test and 5 min cool down (total 130 min, confirmed by coach and captain of soft tennis team).

**FIGURE 1:**
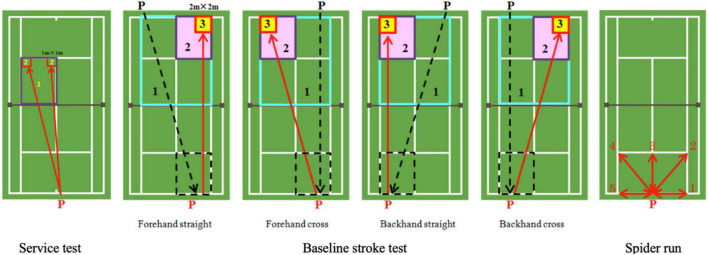
Athletic performance test. Black P is player who strike ball to participant. Red P is participant who strike ball coming from opposite side.

Service performance was tested after warming up. Participants were required not to consider their shots as “first or second serves” but to aim all their serves into the two target boxes across the net (center and side), where the area they could maximize the movement of the opponent were clearly marked with red tape (see Figure 1, left). Participants were asked to serve as accurately as possible 10 times (20 balls in total). All scores were recorded and calculated as follows: in the target, 2 points; out of target but still in the service area, 1 point; and out of the service area, 0 points. Linesman were set for judge.

For the baseline stroke test, participants were instructed to aim at target area (2 m × 2 m) across the net, which was clearly marked with red tape and strike a ball coming from their opposite side (Figure 1, middle). Participants took turns to feed balls to each other, in accurate balls, where those out of the black dotted square were invalid, the participant was fed one more ball instead. Every participant stroked 40 balls (4 conditions: forehand straight = 10, forehand cross = 10, backhand straight = 10, backhand cross = 10). Left and right-handed players both stroked balls under these conditions. Participants’ baseline stroke scores were calculated as follows: in the target area, 3 points; out of the target but on the same side between baseline and service line, 2 points; out of 2-point area but in the opposite single court, 1 point; and out of the opposite single court, 0 points.

Spider run is a tennis-specific sprint test (also use in soft tennis), which was developed by the Japan Tennis Association (2005; Figure 1, right). It measures the on-court movement of the athlete, including speed, coordination and the ability to accelerate and brake over short distances, in five directions and in three different stances, which is used as an essential agility index of players’ on-court performance (Kuroda et al., 2015, 2017; Dobos et al., 2021). Participants were requested to run from the center of the baseline to a marked point and back; then, they would repeat this until five different directions were completed. The total time taken to run to each point and return to the center mark was recorded.

#### Statistical Analyses

All observed values are displayed as means ± standard deviation values. The Shapiro–Wilk test was used to examine the normal distribution of all indices. All data showed a normal distribution except baseline stroke score. To examine the correlation of sleep and performance over the long term, sleep and athletic performance indices were examined after 50 days, we using Pearson’s correlation to analysis data showed normal distribution and Spearman’s rank correlation to analysis data showed non-normal distribution. Considering that the athletic performance test is confounded by the soft tennis experience and sex, partial correlation analyses were also performed. All statistical analyses were conducted using R Studio software version 1.3.1056, and significance was set at *p* < 0.05. Since previous studies designed to examine the association of sleep duration with tennis performance had not reported the effect size, we could not calculate the adequate sample size *a priori*. The current sample size was set based on the previous studies (*N* ≥ 12; Reyner and Horne, 2013; Schwartz and Simon, 2015). Furthermore, the *post hoc* power analyses were conducted to determine statistical power using G*Power 3.1 (Faul et al., 2007, 2009). The effect size was defined as small, medium, and large when *r* = 0.1, 0.3, and 0.5, respectively (Cohen, 2013).

## Results

### Characteristics of Sleep Duration, and Inconsistency

Characteristics of sleep duration and soft tennis athletic performance scores are summarized in Table 1.

**TABLE 1:** Summary of participants’ characteristics, sleep duration and athletic performance test score.

No.	50 days Sleep			Service	Baseline stroke	Spider run
	duration (h)			score	score	time (s)
1	7.6	±	2.8	10	55	18.7
2	6.0	±	1.4	15	42	18.3
3	6.2	±	1.7	14	61	19.1
4	7.6	±	1.2	8	46	19.0
5	7.4	±	1.9	8	54	18.0
6	8.0	±	1.6	16	49	18.1
7	6.7	±	1.2	15	45	18.8
8	7.3	±	1.5	4	58	17.0
9	8.0	±	1.3	12	48	17.8
10	7.1	±	1.9	9	56	18.9
11	7.6	±	0.5	16	55	19.3
12	7.3	±	1.7	3	39	17.9
13	7.8	±	2.0	7	40	18.7
14	9.0	±	2.7	6	46	20.0
15	7.2	±	1.8	5	18	20.4
Average	7.4	±	1.7	9.9 ± 4.5	47.5 ± 10.6	18.7 ± 0.9

*Mean (standard deviation).*

**FIGURE 2:**
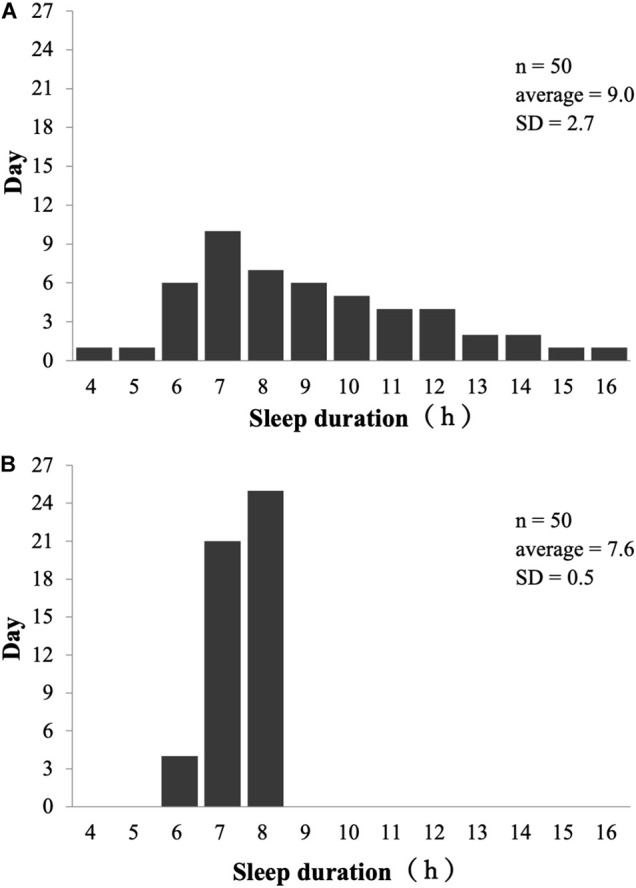
Sleep inconsistency on typical players. **(A)** Example of sleep duration in large variations; **(B)** Example of sleep duration in small variations.

The sleep diary showed varied length during the investigation period of sleep duration (7.4 ± 1.7 h) even in the same person. A typical example of the distribution of sleep duration is presented in Figures 2A,B. The participant with the largest standard deviation of sleep duration slept for 9 h on average with a wide dispersion (mean sleep time = 9.0 h, range (shortest-longest) = 4–16 h, SD = 2.7 h; Figure 2A). In contrast, the participant with small standard deviation of sleep duration slept 7.6 h on average with a narrow dispersion (mean sleep time = 7.6 h, range (shortest-longest) = 6–8 h, SD = 0.5 h; Figure 2B).

### Main Findings

The correlations between sleep index scores and athletic performance are summarized in Table 2.

**TABLE 2:** Correlations and partial correlation (after controlled soft tennis experience and sex) of Sleep Index and athletic performance.

Sleep diary	Sleep index	Service score		Baseline stroke score		Spider run time	
		*Pearson*	*p*	*Spearman*	*p*	*Pearson*	*p*
50 days	Correlation						
	Average sleep duration	−0.317	0.250	−0.038	0.894	0.146	0.604
	SD of sleep duration	−0.452	0.091	0.018	0.950	0.193	0.490
	Partial correlation						
	Average sleep duration	−0.239	0.431	0.116	0.704	0.068	0.825
	SD of sleep duration	−0.562	0.046*	−0.036	0.908	0.266	0.379

*p < 0.05.

#### Relationship Between Sleep Duration and Athletic Performance

There was no significant relationship between mean sleep duration and athletic performance (service score: *r* = −0.32, *p* = 0.25; baseline stroke score: *r*_s_ = −0.04, *p* = 0.89; Spider run time: *r* = 0.15, *p* = 0.60). These results were unchanged after controlling for tennis experience and sex.

#### Relationship Between Sleep Inconsistency and Athletic Performance

**FIGURE 3:**
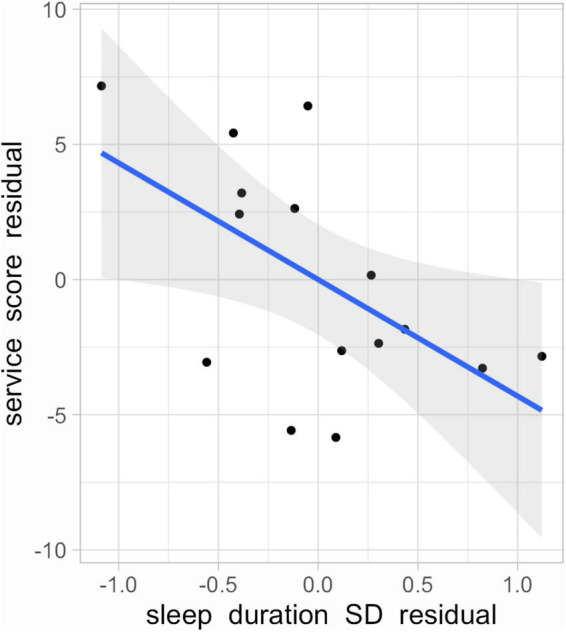
Relationship of sleep duration SD residual and service score residual (*r* = −0.56, *p* = 0.046, 1-β = 0.63).

The inconsistency in sleep duration (SD of sleep duration) was inversely correlated with service score (*r* = −0.45, *p* = 0.09) and this correlation became slightly stronger after controlling for soft tennis experience and sex (*r* = −0.56, *p* = 0.046, 1-β = 0.63, Figure 3). There was no significant relationship between sleep inconsistency and other athletic performance (baseline stroke score: r_s_ = 0.02, *p* = 0.95; Spider run time: *r* = 0.19, *p* = 0.49). These results were unchanged after controlling for tennis experience and sex.

## Discussion

### Main Analyses and Implications

The purpose of this study was to evaluate the relationship between daily sleep status and athletic performance among 15 healthy collegiate soft tennis players. As we hypothesized, the current results showed a tendency that the greater sleep inconsistency showed lower service performance. Regular sleep duration appears to be a possible contributor to improving soft tennis service performance.

### Sleep Duration and Athletic Performance

As compared with sleep extension experiments (Mah et al., 2011; Schwartz and Simon, 2015) which measured the effects among participants who slept 9–10 h every night, the average sleep duration in this study was 7.4 ± 1.7 h. This result is close to collegiate student-athletes at Stanford University: 7.1 ± 1.6 h on campus, 7.6 ± 1.7 h when travelling (Mah et al., 2018), and 7.5 ± 1.1 h of the Italian Tennis Federation control condition group (Vitale et al., 2021), but around 2.8 h less than in the basketball players’ sleep extension experiment (Mah et al., 2011) (10.4 ± 1.1 h) and 1.2 h less than in the tennis players’ sleep extension experiment (Schwartz and Simon, 2015) (8.8 ± 0.6 h). In contrast, compared to the two sleep restriction experiments of tennis players (Reyner and Horne, 2013; Vitale et al., 2021), the average sleep duration in this study was 2.6 and 2.8 h longer respectively.

Although better sleep may reduce the risk of injury and potentially enhance performance, the above-mentioned research suggests that sleep duration was not commensurate with what players optimally required. In fact, quite a number of collegiate athletes fail to obtain the recommended amount of sleep or ideal sleep habits because of the irregular academic, competition schedules, and mental stress in daily life (Watson, 2017; Mah et al., 2018). Our sleep data was naturalistic (recorded without any external manipulation), record period was long (50 days), sleep duration showed big difference even in the same participant, which may explain why we revealed no relationship between average sleep duration and soft tennis competitive performance.

### Inconsistent Sleep Duration and Soft Tennis Athletic Performance

As demonstrated in two previous studies, sleep restriction did lead to a decrease in tennis serve accuracy (Reyner and Horne, 2013; Vitale et al., 2021). In the present study, it is difficult to compare serve accuracy directly because of the different shapes and sizes of the accuracy test area. However, daily sleep inconsistency may lead to a decreased tendency of tennis service accuracy. Despite this, we did not impose any restrictions on sleep duration, and no participants were extreme morning or evening chronotypes as in a previous study (Vitale et al., 2021).

Kirschen et al. (2020) conducted a systematic review on the relationship between sleep duration, sleep quality, and objective athletic performance among competitive athletes across 19 studies, representing 12 sports. They determined that the sports requiring speed, tactical strategy, and technical skill were the most sensitive to sleep duration manipulations. Moreover, another study demonstrated that “social jetlag” may affect athletes’ specific brain areas—those involved in posture control, such as the thalamus and the prefrontal cortex as well as the cerebellum, resulting in poorer performance (Umemura et al., 2018).

Homeostatic sleep drive and the circadian system work together to enhance stable patterns of sleep and wakefulness, in normal sleepers; adherence to regular bedtimes and wake times promotes optimal sleep propensity and consolidation, thus, regular sleep-wake patterns may facilitate a sleep upgrading process (Dijk and Czeisler, 1995; Knufinke et al., 2018a). However, inconsistent sleep may disorganize the synchrony between physiological sleep drive, circadian rhythms, and the nocturnal sleep episode (Stepanski and Wyatt, 2003), reduce wakefulness and lead to lower performance in competition.

Knufinke et al. (2018a) analyzed 98 highest (inter-)national level elite athletes’ non-manipulated sleep, psychomotor vigilance and performance data on three non-consecutive nights within a 7 day monitoring period. Their results indicated natural variation in sleep quantity impacts psychomotor vigilance to a greater extent than athletic performance and suggested one night minor sleep loss may not immediately have negative impacts, but extreme sleep loss or accumulated sleep debt, may have more severe consequences.

Furthermore, unlike a service, which a player can complete by him/herself after deliberate consideration, an effective baseline stroke tests athletes’ ability to estimate the speed and direction of the coming ball, analyze spin level, and then stroke the ball back all in a short time. These variables are further influenced by different opponents, which could be one reason for our finding no relationship between sleep duration, inconsistency and baseline stroke score. Although it is clear that sleep deprivation generates negative effects such as crosscourt stroke accuracy (Vitale et al., 2021), reaction time, and cognitive function (Taheri and Arabameri, 2012), daily sleep inconsistency seems not to have the same effect.

### Limitations

First, sleep data in the present study were self-reported; therefore, it is difficult to evaluate participants’ sleep condition objectively. Second, due to the different shapes and sizes of the accuracy test area, it was difficult for us to directly compare the accuracy of serves (Reyner and Horne, 2013; Schwartz and Simon, 2015; Vitale et al., 2021). Speed of service and baseline stroke speed was not measured; thus, participants may have focused more on accuracy than on speed or power to improve their test score. Third, only 15 participants persisted complete 50 days sleep diary, only 2 were female and none of them were a top-level soft tennis player. In the future, it is necessary to verify with rigorous, larger samples including more females. Fourth, we found that the greater sleep inconsistency may be associated with impaired serve performance, and the effect size was large (*r* = −0.56). However, the *post hoc* power analysis showed a statistical power of 63%. Fifth, diurnal naps are regarded as an advantageous intervention to enhance the recovery process and mitigate the negative effect of partial sleep deprivation on physical and cognitive performance (Lastella et al., 2021; Souabni et al., 2021). In the present study, although nap records were found in several players’ sleep diaries, they were not habitual, so it was difficult for us to make further analyses on naps. Sixth, due to the flexible training schedule and timetable changing in 50-day record time (from summer vacation to autumn semester), it was difficult to assess the weekly training time of every participant. To enhance reliability, a similar experiment with varied athletes, involving different training types, night match and top-level players are recommended. Finally, psychological, training, and living habitual factors like mental health, late-evening consumption of heavy meals, night games, common use of electric devices and amber lenses should be considered in the future (Burkhart and Phelps, 2009; Halson, 2016; Romyn et al., 2016; Knufinke et al., 2018a; Nedelec et al., 2018; Vitale et al., 2019; Bonato et al., 2020).

### Conclusion

This study evaluated the relationship between long-term daily sleep duration and competitive performance among 15 healthy collegiate soft tennis players. The results revealed that inconsistency in sleep duration (SD of sleep duration) showed a tendency with service score (*r* = −0.45, *p* = 0.09), and this correlation became slightly stronger after controlling for soft tennis experience and sex (*r* = −0.56, *p* = 0.046). This indicates the key role of regular sleep for athletes’ service, despite the under-recommended sleep duration of our participants. In sum, athletes who get regular sleep in the long term may perform better in service than those who get varied amounts of sleep.

## Data Availability Statement

The original contributions presented in the study are included in the article/supplementary material, further inquiries can be directed to the corresponding author/s.

## Ethics Statement

The studies involving human participants were reviewed and approved by Hokkaido University Faculty of Education Ethics Committee (No. 17-37-02). The participants provided their written informed consent to participate in this study.

## Author Contributions

TH and MM participated in the design of the study and contribute to data collection and data analysis. WW contributed to tennis competitive performance data collection. YK contributed to the tennis competitive performance test design and interpretation of the results. All authors contributed to the article and approved the submitted version.

## Conflict of Interest

The authors declare that the research was conducted in the absence of any commercial or financial relationships that could be construed as a potential conflict of interest.

## Publisher’s Note

All claims expressed in this article are solely those of the authors and do not necessarily represent those of their affiliated organizations, or those of the publisher, the editors and the reviewers. Any product that may be evaluated in this article, or claim that may be made by its manufacturer, is not guaranteed or endorsed by the publisher.

## References

[B1] Bonato M. La Torre A. Marventano I. Saresella M. Merati G. Banfi G. (2020). Effect of high-intensity interval training versus small-sided games training on sleep and salivary cortisol level. *Int. J. Sports Physiol. Perform.* 2020 1–8. 10.1123/ijspp.2019-0498 32871556

[B2] Burkhart K. Phelps J. R. (2009). Amber lenses to block blue light and improve sleep: a randomized trial. *Chronobiol. Int.* 26 1602–1612. 10.3109/07420520903523719 20030543

[B3] Cohen J. (2013). *Statistical power analysis for the Behavioral Science.* Cambridge, MA: Academic Press.

[B4] Dijk D. J. Czeisler C. A. (1995). Contribution of the circadian pacemaker and the sleep homeostat to sleep propensity, sleep structure, electroencephalographic slow waves, and sleep spindle activity in humans. *J. Neurosci.* 15 3526–3538. 10.1523/JNEUROSCI.15-05-03526.1995 7751928 PMC6578184

[B5] Dobos K. Novak D. Barbaros P. (2021). Neuromuscular fitness is associated with success in sport for elite female, but not male tennis players. *Int. J. Environ. Res. Public Health* 18:6512. 10.3390/ijerph18126512 34204221 PMC8296339

[B6] Faul F. Erdfelder E. Buchner A. Lang A. G. (2009). Statistical power analyses using G*Power 3.1: Tests for correlation and regression analyses. *Behav. Res. Methods* 41 1149–1160. 10.3758/BRM.41.4.1149 19897823

[B7] Faul F. Erdfelder E. Lang A. G. Buchner A. (2007). G*Power 3: A flexible statistical power analysis program for the social, behavioral, and biomedical sciences. *Behav. Res. Methods* 39 175–191. 10.3758/bf03193146 17695343

[B8] Filipas L. Ferioli D. Banfi G. La Torre A. Vitale J. A. (2021). Single and combined effect of acute sleep restriction and mental fatigue on basketball free-throw performance. *Int. J. Sports Physiol. Perform.* 16 415–420. 10.1123/ijspp.2020-0142 33440343

[B9] Halson S. L. (2016). Stealing sleep: Is sport or society to blame? *Br. J. Sports Med.* 50 381–381. 10.1136/bjsports-2015-094961 26612841

[B10] Horne J. A. Ostberg O. (1976). A self-assessment questionnaire to determine morningness-eveningness in human circadian rhythms. *Int. J. Chronobiol.* 4 97–110. 1027738

[B11] Irish L. A. Kline C. E. Gunn H. E. Buysse D. J. Hall M. H. (2015). The role of sleep hygiene in promoting public health: A review of empirical evidence. *Sleep Med. Rev.* 22 23–36. 10.1016/j.smrv.2014.10.001 25454674 PMC4400203

[B12] Janse Van Rensburg D. C. Jansen Van Rensburg A. Fowler P. M. Bender A. M. Stevens D. Sullivan K. O. (2021). Managing travel fatigue and jet lag in athletes: A review and consensus statement. *Sports Med.* 51 2029–2050. 10.1007/s40279-021-01502-0 34263388 PMC8279034

[B13] Kirschen G. W. Jones J. J. Hale L. (2020). The Impact of Sleep Duration on Performance Among Competitive Athletes: A Systematic Literature Review. *Clin. J. Sport Med.* 30 503–512. 10.1097/JSM.0000000000000622 29944513

[B14] Knufinke M. Nieuwenhuys A. Maase K. Moen M. H. Geurts S. A. E. Coenen A. M. L. (2018b). Effects of natural between-days variation in sleep on elite athletes’ psychomotor vigilance and sport-specific measures of performance. *J. Sports Sci. Med*. 17 515–524. 30479518 PMC6243629

[B15] Knufinke M. Nieuwenhuys A. Geurts S. A. E. Coenen A. M. L. Kompier M. A. J. (2018a). Self-reported sleep quantity, quality and sleep hygiene in elite athletes. *J. Sleep Res.* 27 78–85. 10.1111/jsr.12509 28271579

[B16] Kuroda Y. Suzuki N. Dei A. Umebayashi K. Takizawa K. Mizuno M. (2015). A comparison of the physical fitness, athletic performance and competitive achievements of junior and senior tennis players. *MoHE* 4:43. 10.15282/mohe.v4i0.43

[B17] Kuroda Y. Takizawa K. Mizuno M. (2017). Relevance of perceived exertion and accuracy of second serve in collegiate mens tennis players. *J. Sport Hum. Perform.* 4 1–10. 10.12922/jshp.v4i4.92

[B18] Lastella M. Halson S. L. Vitale J. A. Memon A. R. Vincent G. E. (2021). To nap or not to nap? A systematic review evaluating napping behavior in athletes and the impact on various measures of athletic performance. *Nat. Sci. Sleep* 13 841–862. 10.2147/NSS.S315556 34194254 PMC8238550

[B19] Mah C. D. Kezirian E. J. Marcello B. M. Dement W. C. (2018). Poor sleep quality and insufficient sleep of a collegiate student-athlete population. *Sleep Health* 4 251–257. 10.1016/j.sleh.2018.02.005 29776619

[B20] Mah C. D. Mah K. E. Kezirian E. J. Dement W. C. (2011). The effects of sleep extension on the athletic performance of collegiate basketball players. *Sleep* 34 943–950. 10.5665/SLEEP.1132 21731144 PMC3119836

[B22] Nedelec M. Aloulou A. Duforez F. Meyer T. Dupont G. (2018). The variability of sleep among elite athletes. *Sports Med. Open*. 4:34. 10.1186/s40798-018-0151-2 30054756 PMC6063976

[B23] Okano K. Kaczmarzyk J. R. Dave N. Gabrieli J. D. E. Grossman J. C. (2019). Sleep quality, duration, and consistency are associated with better academic performance in college students. *npj Sci. Learn.* 4:16. 10.1038/s41539-019-0055-z 31583118 PMC6773696

[B24] Reyner L. A. Horne J. A. (2013). Sleep restriction and serving accuracy in performance tennis players, and effects of caffeine. *Physiol. Behav.* 120 93–96. 10.1016/j.physbeh.2013.07.002 23916998

[B25] Romyn G. Robey E. Dimmock J. A. Halson S. L. Peeling P. (2016). Sleep, anxiety and electronic device use by athletes in the training and competition environments. *Eur. J. Sport Sci.* 16 301–308. 10.1080/17461391.2015.1023221 25790844

[B26] Schwartz J. Simon R. D. (2015). Sleep extension improves serving accuracy: a study with college varsity tennis players. *Physiol. Behav.* 151 541–544. 10.1016/j.physbeh.2015.08.035 26325012

[B27] Souabni M. Hammouda O. Romdhani M. Trabelsi K. Ammar A. Driss T. (2021). Benefits of daytime napping opportunity on physical and cognitive performances in physically active participants: A systematic review. *Sports Med.* 51 2115–2146. 10.1007/s40279-021-01482-1 34043185

[B28] Stepanski E. J. Wyatt J. K. (2003). Use of sleep hygiene in the treatment of insomnia. *Sleep Med. Rev.* 7 215–225. 10.1053/smrv.2001.0246 12927121

[B29] Taheri M. Arabameri E. (2012). The effect of sleep deprivation on choice reaction time and anaerobic power of college student athletes. *Asian J. Sports Med.* 3 15–20. 10.5812/asjsm.34719 22461961 PMC3307962

[B30] Umemura G. S. Pinho J. P. Goncalves B. D. B. Furtado F. Forner-Cordero A. (2018). Social jetlag impairs balance control. *Sci. Rep.-Uk* 20:8. 10.1038/s41598-018-27730-5 PMC6010412 29925863

[B31] Vitale J. A. Banfi G. Galbiati A. Ferini-Strambi L. La Torre A. (2019). Effect of a night game on actigraphy-based sleep quality and perceived recovery in top-level volleyball athletes. *Int. J. Sports Physiol. Perform.* 14 265–269. 10.1123/ijspp.2018-0194 30040006

[B32] Vitale J. A. Bonato M. Petrucci L. Zucca G. La Torre A. Banfi G. (2021). Acute sleep restriction affects sport-specific but not athletic performance in junior tennis players. *Int. J. Sports Physiol. Perform.* 2021 1–6. 10.1123/ijspp.2020-0390 33607625

[B33] Watson A. M. (2017). Sleep and athletic performance. *Curr. Sports Med. Rep.* 16 413–418. 10.1249/JSR.0000000000000418 29135639

[B34] Yamanaka Y. Hashimoto S. Tanahashi Y. Nishide S. Honma S. Honma K. (2010). Physical exercise accelerates reentrainment of human sleep-wake cycle but not of plasma melatonin rhythm to 8-h phase-advanced sleep schedule. *Am. J. Physiol. Regul. Integr. Comp. Physiol.* 298 R681–R691. 10.1152/ajpregu.00345.2009 20042689

