# Corrigendum: The relationships of sleep duration and inconsistency with the athletic performance of collegiate soft tennis players

**DOI:** 10.3389/fpsyg.2023.1237347

**Published:** 2023-07-07

**Authors:** Tianfang Han, Wenjuan Wang, Yuta Kuroda, Masao Mizuno

**Affiliations:** ^1^Graduate School of Health Sciences, Hokkaido University, Sapporo, Japan; ^2^Graduate School of Education, Hokkaido University, Sapporo, Japan; ^3^Department of Sport Education, Hokusho University, Ebetsu, Japan; ^4^Faculty of Education, Hokkaido University, Sapporo, Japan; ^5^Faculty of Health and Medical Care, Hachinohe Gakuin University, Hachinohe, Japan

**Keywords:** sleep duration, sleep inconsistency, serve, performance, agility

In the published article, there was an error in Discussion, *Inconsistent Sleep Duration and Soft Tennis Athletic Performance*, Paragraph 5. The paragraph previously stated “Although it is clear that sleep deprivation generates negative effects such as serve accuracy (Reyner and Horne, 2013),” but this should be “Although it is clear that sleep deprivation generates negative effects such as crosscourt stroke accuracy (Vitale et al., 2021),”. The corrected paragraph appears below.

Furthermore, unlike a service, which a player can complete by him/herself after deliberate consideration, an effective baseline stroke tests athletes' ability to estimate the speed and direction of the coming ball, analyze spin level, and then stroke the ball back all in a short time. These variables are further influenced by different opponents, which could be one reason for our finding no relationship between sleep duration, inconsistency and baseline stroke score. Although it is clear that sleep deprivation generates negative effects such as crosscourt stroke accuracy (Vitale et al., 2021), reaction time, and cognitive function (Taheri and Arabameri, 2012), daily sleep inconsistency seems not to have the same effect.

In addition there was an error in the Correspondence section as published. The corresponding author's email was incorrect. The email should be tflunwen@gmail.com.

In the published article, there was also an error in the Acknowledgments statement as published. An acknowledgment to Dr. Yujiro Yamanaka was erroneously excluded. The revised acknowledgments statement appears below.
