# Corrigendum: The relationships of sleep duration and inconsistency with the athletic performance of collegiate soft tennis players

**DOI:** 10.3389/fpsyg.2024.1500832

**Published:** 2024-10-21

**Authors:** Tianfang Han, Wenjuan Wang, Yuta Kuroda, Masao Mizuno

**Affiliations:** ^1^Graduate School of Health Sciences, Hokkaido University, Sapporo, Japan; ^2^Graduate School of Education, Hokkaido University, Sapporo, Japan; ^3^Department of Sport Education, Hokusho University, Ebetsu, Japan; ^4^Faculty of Education, Hokkaido University, Sapporo, Japan; ^5^Faculty of Health and Medical Care, Hachinohe Gakuin University, Hachinohe, Japan

**Keywords:** sleep duration, sleep inconsistency, serve, performance, agility

In the published article, there was an error in Figure 2 *Sleep inconsistency on typical players* as published. We found that the average sleep duration (h) and standard deviation (SD) of the two players marked in Figure 2 were incorrectly reversed. The corrected Figure 2 and its caption appear below.

**Figure 2:**
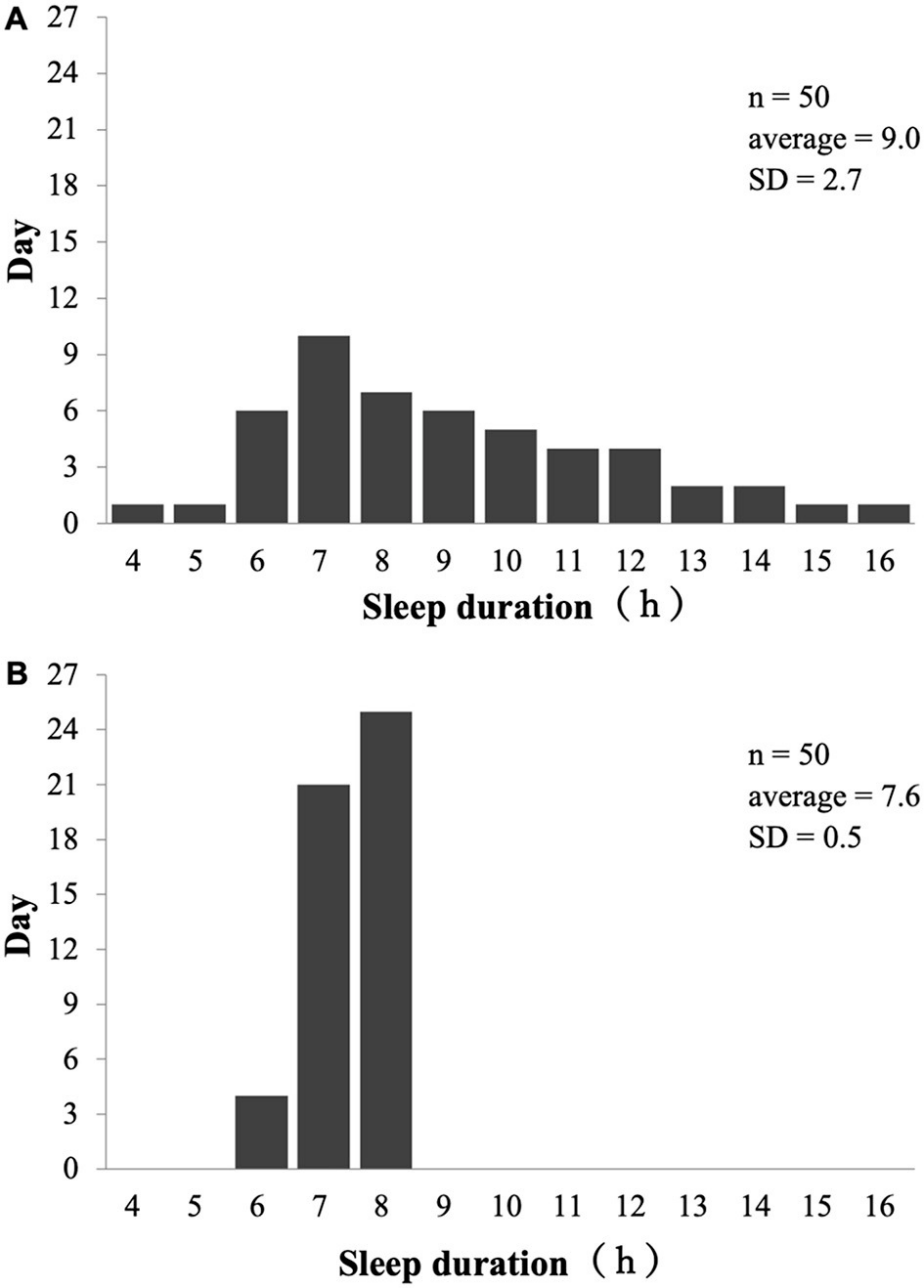
Sleep inconsistency on typical players. **(A)** Example of sleep duration in large variations; **(B)** Example of sleep duration in small variations.

The authors apologize for this error and state that this does not change the scientific conclusions of the article in any way. The original article has been updated.

